# The application of PRECIS-2 ratings in randomized controlled trials of Chinese herbal medicine

**DOI:** 10.18632/oncotarget.22204

**Published:** 2017-10-31

**Authors:** Liming Lu, Li Zhou, Jing Dong, Yu Xiang, Zehuai Wen

**Affiliations:** ^1^ The Second Affiliated Hospital of Guangzhou University of Chinese Medicine, Guangdong Provincial Hospital of Chinese Medicine, Guangzhou, China; ^2^ National Center for Design Measurement and Evaluation in Clinical Research, Guangzhou University of Chinese Medicine, Guangzhou, China

**Keywords:** pragmatic-explanatory continuum indicator summary (PRECIS), Chinese herbal medicine, comparative effectiveness research (CER), randomized controlled trials (RCTs)

## Abstract

This study tests the feasibility of applying the pragmatic-explanatory continuum indicator summary (version “PRECIS-2”) tool to randomized controlled trials of Chinese herbal medicine. A search was conducted to identify potentially eligible randomized controlled trials. Using the PRECIS-2 tool, assessment of trials was performed independently by 2 evaluators using a scale of 1–5 for each criterion (1 = maximal efficacy, 5 = maximal effectiveness). A total of 7,166 reports were retrieved from databases and 159 were included in the full text. Though PRECIS-2 describes quantitative scoring in detail, evaluators were uncertain about several specific operationalizations and found high evaluator variation in the first independent ratings. After discussion and reaching consensus, inter-evaluator reliability improved. For PRECIS-2 ratings over time, there was no evidence that the design and performance of RCTs of CHM paid more attention to “efficacy” criteria after the implementation of PRECIS (all *P* > 0.05). More research is needed to establish the easiest and most useful tool to distinguish between effectiveness and efficacy results.

## INTRODUCTION

From a comparative effectiveness research (CER) perspective, the “effectiveness” of an intervention in pragmatic trials refers to the extent to which it benefits the targeted population in routine circumstances, with the goal of supporting informed decision-making and improving healthcare. [1–3] By contrast, the “efficacy” of an intervention is related to the degree to which the intervention does what is intended under ideal conditions by means of an explanatory randomized controlled trial (RCT). [4, 5] Nowadays, many investigators do not value or distinguish between these two concepts when designing and performing clinical trials. They apparently choose these terms randomly, often neglecting the study’s true purpose. [6]

The pragmatic-explanatory continuum indicator summary (PRECIS) tool which was developed in 2009 [7] and improved in 2015 (version “PRECIS-2”), [8] has been designed to help researchers distinguish between effectiveness and efficacy issues at the design stage of a trial. It also helps ensure that their design options are consistent with their purpose. [8] PRECIS-2 has nine domains, each scored on a 5-point Likert continuum (from 1 = maximal efficacy to 5 = maximal effectiveness). This benefits designers by allowing them to determine whether design options meet their intended purpose in critical appraisal. It can also be used for systematic review, funding, ethics, and publication decisions on RCTs. [8] It is important to note that there is no trial of pure effectiveness or pure efficacy, and different weights exist for terms in a continuum for different features of the trial design.

CER is particularly valuable for interventions with high variation in practices widely used in daily life. [9, 10] Chinese medicine (CM) is becoming increasingly prevalent in Europe and North America and has some variation in diagnoses and treatment (e.g. syndrome differentiation). [11–13] In RCTs of Chinese medicine, it is difficult for a researcher to discriminate and execute measures of “efficacy” and “effectiveness” in accordance with the purpose. Thus, our study aims to 1) test the feasibility of applying the PRECIS tool to RCTs of Chinese herbal medicine (CHM); 2) evaluate the extent to which RCTs of CHM are explanatory using PRECIS-2 efficacy as the goal of RCTs.

## RESULTS

### Search results

**Figure 1:**
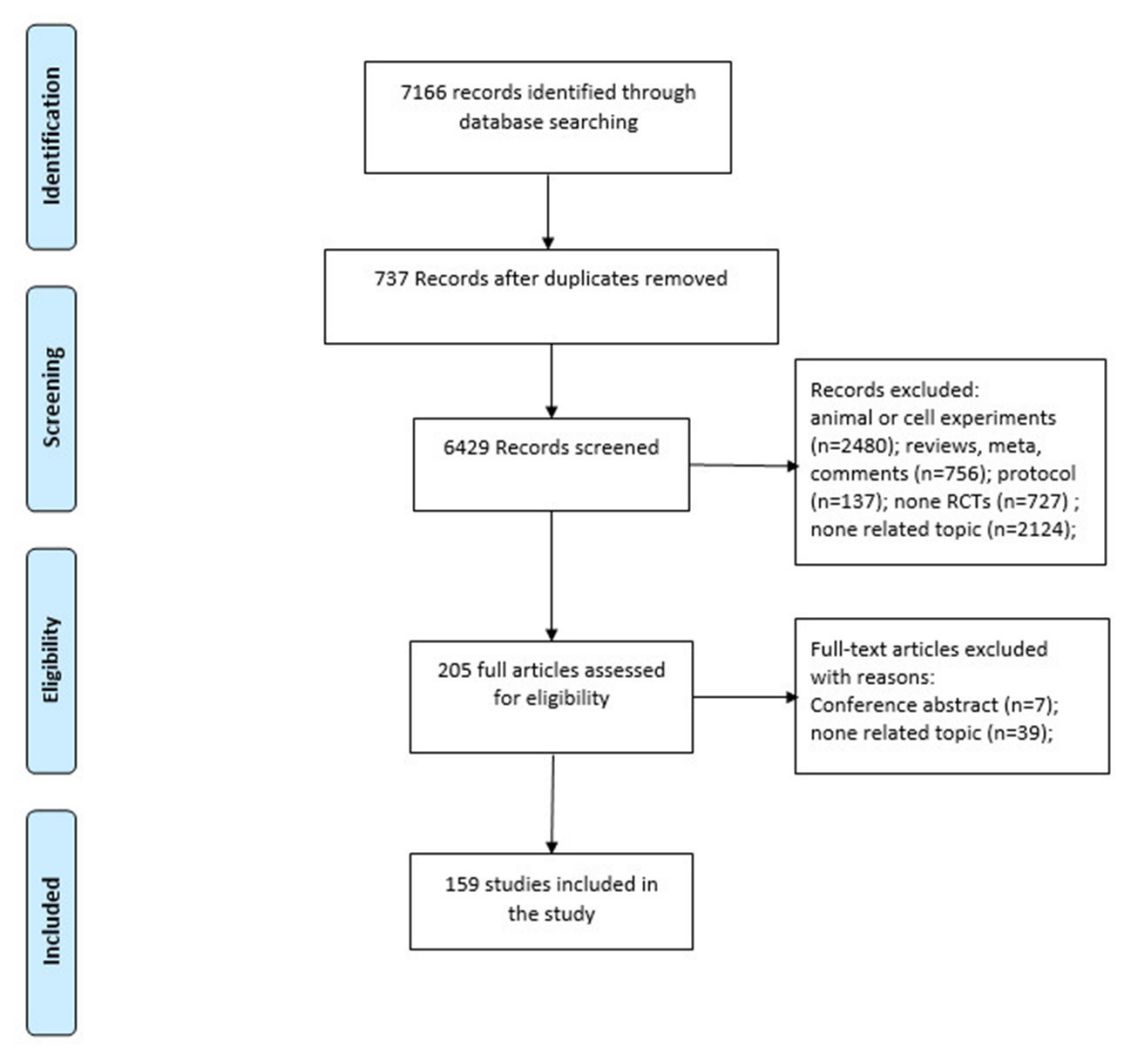
Article selection process

7,166 unique citations were retrieved: 913 from Medline, 2,753 from Embase, 1,669 from AMED and 1,831 from CENTRAL. Of these, 6,961 were excluded after identification, screening and eligibility processes, based on titles or abstracts, leaving 1,289 for full-text review. Of the 205 remaining full citations, 46 were excluded and 159 were included. The selection process for all articles is presented in Figure 1.

### Characteristics

**Figure 2:**
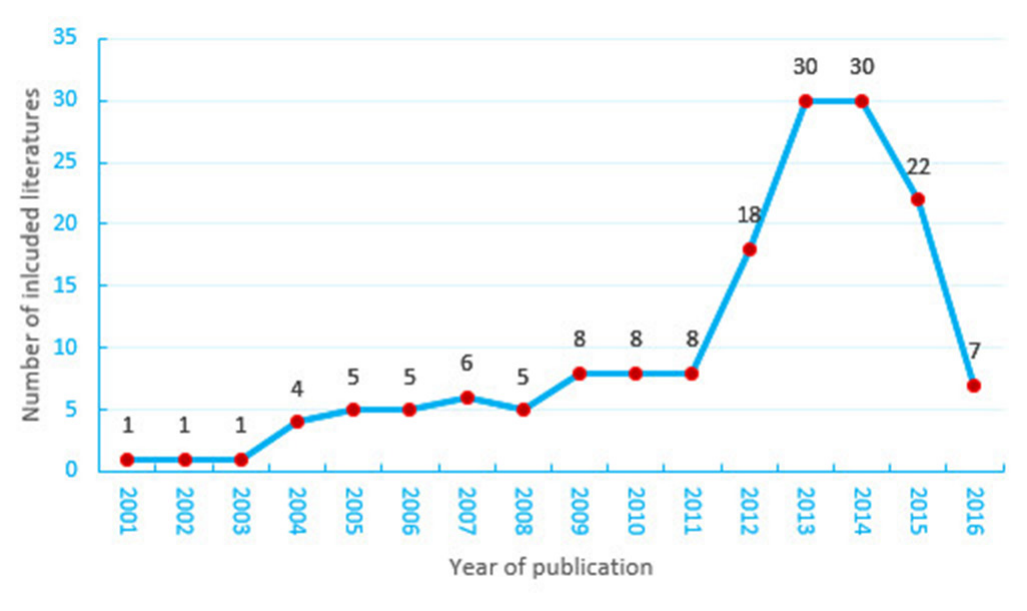
Randomized controlled trials of Chinese herbal medicine published in English between 2001 and 2016

Characteristics of the 159 selected RCTs are presented in Table 1. The frequency of RCTs of CHM was found to have increased most of the time, but to have declined in 2015 (Figure 2). Table 1 shows that the most common CHM formulations were capsules (53, 33.3%), granules (29, 18.2%) and tablets (17, 10.7%). The digestive system (29, 18.2%) and the nervous system (27, 17.0%) were each major research areas. The majority of the RCTs were conducted in multiple centers (84, 52.8%) and in mainland China (98, 61.6%), with 2 study groups (129, 81.1%) and funding (137, 86.2%). The average sample size was 120, and the majority of the RCTs had been published in journals with impact factors in the range of 1.93-3.0.

**Table 1:** Characteristics of included RCTs

Features of included RCTs	No. of studies	%
Study groups		
2	129	81.1
3	24	15.1
4	5	3.1
5	1	0.6
Sample size		
Median (range)	120 (16-3505)	100.0
Study center		
Single center	71	44.7
Multi-center	84	52.8
Unclear	4	2.5
Impact factor		
Mainland China	1.93	61.6
Taiwan	1.93	6.3
South Korea	3.0	6.3
USA	2.95	5.7
Iran	2.57	4.4
Hong Kong	2.24	3.8
Japan	1.93	3.1
Bodily systems		
Motion system	11	6.9
Digestive system	29	18.2
Respiratory system	19	11.9
Urinary system	5	3.1
Reproductive system	12	7.5
Endocrine System	21	13.2
Immune System	13	8.2
Nervous system	27	17.0
Circulatory system	22	13.8
Sources of trial funding		
No funding	22	13.8
International funding	4	2.5
National funding	66	41.5
Provincial funding	33	20.8
Regional funding	1	0.6
Funding from university or work unit	22	13.8
Funding from company	11	6.9
Formulation of Chinese herbal medicine		
Granules	29	18.2
Decoction	15	9.4
Oral liquid	2	1.3
Extract	4	2.5
Capsule	53	33.3
Injection	1	0.6
Herbal tea	2	1.3
Pill	5	3.1
Powder	13	8.2
Ointment	5	3.1
Tablet	17	10.7
Other	13	8.2

Abbreviations: RCTs, randomized controlled trials; SD, standard deviation.

### Inter-evaluator reliability of ratings

As shown in Table 2, evaluators believed that good judgement existed in the criteria of “Eligibility” and “Organisation,” while moderate judgement existed in other criteria.

**Table 2:** Rating details of the efficacy-effectiveness continuum

Criteria	Rating# max. diff. points	Intraclass correlation before/after∆	Operationalization good/moderate/difficult	Comments
1. Eligibility	1	0.67/0.89	good	The PRECIS-2^*^ tool provides clear guidelines for evaluators to judge the difference in patients with usual care and ideal care
2. Recruitment	1	0.28/0.89	moderate	Evaluators are unclear on determining what counts as usual care, especially, when the trial does not report any recruitment information.
3. Setting	2	0.42/0.57	moderate	Sometimes, the evaluators are confused about how to identify “specialist, academic centres” or hospitals.
4. Organisation	0	0.44/1.0	good	It is easy for us to judge this item.
5.Flexibility (delivery)	1	0.20/0.93	moderate	Sometimes, publications do not provide enough information about “a highly specified, protocol driven intervention,” the compliance, restrictions or side effects. It is difficult to determine the extent (scoring 1 or 2 is vague).
6.Flexibility (adherence)	1	0.49/0.58	moderate	As mentioned above, scoring 1 or 2 is vague about whether all proposed requirements must be satisfied, or just some of them?
7. Follow-up	1	0.68/0.87	moderate	Trial situations always differ from usual care. The evaluators are unsure how to determine “follow-up visits that are more frequent than occur under usual care.”
8.Primary outcome	2	0.42/0.76	moderate	Evaluators need to be well-informed about composite and surrogate outcomes. Publications do not provide enough information about “having central adjudication of the outcome or using an assessment that needs special training or tests not normally used in usual care”.
9. Primary analysis	1	0.46/0.96	moderate	Uncertain judgment exists when older publications do not provide the information related to “intent-to-treat” or “per-protocol” analysis.

#after consensus max difference of points (scale 1–5, 1 = max. efficacy to 5 = max. effectiveness) for each of the trials for this criteria; ∆ Before: before consensus, after: after consensus; ^*^ PRECIS: a pragmatic explanatory continuum indicator summary.

Though PRECIS-2 describes quantitative scoring in detail, evaluators were uncertain about several specific operations and got high evaluator variation in the first independent ratings. Several comments we proposed were as follows: uncertain judgement existed when some key information was not reported; judgement still lacked clear quantitative illustration in some items; it was difficult to determine the extent (scoring 1 or 2 is vague). After consensus agreement, inter-evaluator reliability was improved and most of the differences in judgement between the two evaluators were 1 point.

### PRECIS-2 ratings over time

The differences in the percentage of ratings of the efficacy-effectiveness continuum in studies published before and after the implementation of PRECIS are presented in Table 3. There was no evidence that the design and performance of RCTs of CHM paid more attention to “efficacy” criteria after the implementation of PRECIS (all *P*>0.05).

**Table 3:** PRECIS-2 ratings over time

Item	Criteria	Description	Year^*^	Rating PRECIS criteria (*n*(%))^#^					Median	*P*
				1	2	3	4	5		
1	Eligibility	To what extent are the participants in the trial similar to those who would have received this intervention had it been part of the usual care?	Before	24(24)	69(69)	2(2)	3(3)	2(2)	2	0.061
			After	20(34)	39(66)	0(0)	0(0)	0(0)	2	
2	Recruitment	How much extra effort is made to recruit participants over and above what would be used in the usual care setting to engage with patients?	Before	7(7)	6(6)	57(57)	30(30)	0(0)	3	0.141
			After	4(7)	2(3)	28(48)	25(42)	0(0)	3	
3	Setting	How different are the settings of the trial from the usual care settings?	Before	16(16)	9(9)	6(6)	51(51)	18(18)	4	0.136
			After	5(9)	6(10)	2(3)	31(53)	15(25)	4	
4	Organisation	How different are the resources, provider expertise, and the organisation of care delivery in the intervention arm of the trial from those available in the usual care?	Before	1(1)	9(9)	64(64)	25(25)	1(1)	3	0.189
			After	1(2)	4(7)	32(54)	22(37)	0(0)	3	
5	Flexibility (delivery)	How different is the flexibility in how the intervention is delivered and the flexibility anticipated in usual care?	Before	16(16)	63(63)	12(12)	9(9)	0(0)	2	0.466
			After	18(31)	25(42)	9(15)	7(12)	0(0)	2	
6	Flexibility (adherence)	How different is the flexibility in how participants are monitored and encouraged to adhere to intervention, from the flexibility anticipated in usual care?	Before	18(18)	76(76)	4(4)	1(1)	1(1)	2	0.651
			After	13(22)	42(71)	4(7)	0(0)	0(0)	2	
7	Follow-up	How different is the intensity of measurement and follow-up of participants in the trial from the typical follow-up in usual care?	Before	12(12)	55(55)	30(30)	3(3)	0(0)	2	0.392
			After	4(7)	33(56)	20(34)	2(3)	0(0)	2	
8	Primary outcome	To what extent is the trial’s primary outcome directly relevant to participants?	Before	20(20)	41(41)	5(5)	31(31)	3(3)	2	0.943
			After	11(19)	27(46)	1(2)	18(31)	2(3)	2	
9	Primary analysis	To what extent are all data included in the analysis of the primary outcome?	Before	12(12)	31(31)	14(14)	33(33)	10(10)	3	0.742
			After	6(10)	17(29)	12(20)	16(27)	8(14)	3	

^*^Before: before the year 2013 (including 2013), after: after the year 2013; ^#^scale 1–5, 1 = max. efficacy to 5 = max. effectiveness for each of the trials for this criteria.

## DISCUSSION

This study aimed to analyze RCTs of CHM in order to characterize explanatory versus pragmatic design, and how design details changed before and after the implementation of PRECIS. It was found that after the implementation of PRECIS, the design and performance of RCTs of CHM did not improve, in terms of “efficacy” criteria.

We tested the feasibility of applying the PRECIS tool to appraising the efficacy-effectiveness continuum of RCTs of CHM. Due to insufficient information and lacking clear quantitative illustration of several items, high variation and uncertainty existed in the first independent ratings. Our results were similar to those of previous studies. Witt CM [14] observed that much of the heterogeneity observed between the evaluators was due to information missing from publications or difficulty in operationalizing the criteria. Johnson KE [15] pointed out that evaluators struggled to use the PRECIS system for analysis, as large differences existed in inter-evaluator reliability. Furthermore, El DR et al [16] indicated that the clinical expertise of the investigator also affected scoring in each domain of PRECIS-2.

In our study, evaluators held different understandings and judgements when they referred to illustrations of PRECIS-2 or when there was missing information. For example, it was unclear for evaluators which situation constituted the usual care, especially when the trial did not report any recruitment information; sometimes, the evaluators were confused about how to identify “specialist, academic centres” or hospitals; it was difficult to determine degree since a binary scale of 1 or 2 was utilized; the evaluators were unsure how to determine “follow-up visits that are more frequent than occur under usual care;” publications did not provide enough information about “having central adjudication of the outcome or using an assessment that needs special training or tests not normally used in usual care;” judgements were uncertain when older publications did not provide the information related to “intent-to-treat” or “per-protocol” analysis. The challenges pertaining to using the tool, especially for certain criteria, suggest that the PRECIS-2 criteria need to be further refined in order to achieve specificity sufficient to enable evaluators to perform quantitative judgment.

To assess the impact of the introduction of PRECIS on the design and implementation of RCTs in this field, we used PRECIS-2 to compare the distribution of each criterion, both before and after 2013. Our results illustrated that there was no improvement in “efficacy” criteria after the implementation of PRECIS. The reasons for this are as follows: 1) the promotion of PRECIS and the importance of considering “efficacy” and “effectiveness” criteria before trial design were insufficient; 2) due to language barriers and a lack of instructions for the Chinese version of PRECIS, many Chinese scholars do not notice the discrepancy between “efficacy” and “effectiveness” criteria before preparing RCTs of Chinese herbal medicine; 3) the challenges and variations in the understanding and usage of PRECIS, especially for certain criteria, hampers researchers ability to use it. There were moderate judgements existed in the criteria of “Setting” and “Flexibility (adherence)” between two evaluators. However, evaluators believed that good judgement existed other criteria.

Though some limitations exist in applying PRECIS-2, its utility benefits the capturing of complete trial information and judging whether the design is consistent with research objectives. This enables better comparisons across trials and allows for analysis of a broader trial portfolio. We propose several suggestions: 1) More research is urgently needed to establish the easiest and most useful tool to facilitate the applicability of results in clinical practice, distinguish between effectiveness and efficacy results and assist researchers in preparing and planning clinical trials; [16] 2) Researchers should pay attention to PRECIS-2 before they design RCTs and promote self-review during their implementation. 3) Due to the large number of Chinese researchers, the PRECIS-2 guidelines should be translated into Chinese; Related introductory articles should also be published in Chinese to promote a wider range of applications for PRECIS-2; 4) Journals all over the world that publish clinical trials should require authors to include a quantitative score related to the effectiveness or efficacy of their combined research articles; [16–18] 5) Several issues specific to CHM should be clarified in the new version of PRECIS. For flexibility (delivery), how do we define “a highly specified, protocol driven intervention” and “permitted co-interventions” in CHM, as doctors of Chinese medicine add or subtract herbs based on syndrome differentiation at different times? A special assessment criterion between effectiveness and efficacy in CHM is needed.

## MATERIALS AND METHODS

### Literature search

A search of Medline, Embase, AMED (the Allied and Complementary Medicine Database) and CENTRAL (Cochrane Library) databases from their inception until December 2016 was conducted to identify potentially eligible studies. We used the string (‘‘Chinese herbal drugs” OR “oriental traditional medicine” OR ‘‘east Asian traditional medicine” OR “herbal medicine” OR “herbaceous agent” OR “Chinese adj5 (herb^*^ or medic^*^ or drug^*^)” OR “herb^*^ adj5 (medic^*^ or drug^*^)”). No language restrictions were imposed, and the reference lists of all relevant studies were checked for further reports. The search strategy can be found in the Supplementary Materials.

### Types of studies

RCTs were included which evaluated the effects of Chinese herbal medicines for any disease. Quasi-randomized trials were excluded.

### Types of interventions

Included interventions included: 1) single herb; 2) Chinese proprietary herbal medicine (usually taken as granules, decoction, oral liquid, extract, capsule, injection, herbal tea, pills, powder, ointment, tablets); 3) herbal mixture prescribed by an herbalist (individualized treatment), and usually tailored to an individual’s pattern of symptoms. There were no limits on approval status, formulation or mode of administration for herbal medicines. Studies of integrative medicine were excluded.

### Comparison group

Placebo, treatment as usual, an alternative presentation of interventions of the study group, no treatment or other active interventions were included as the control group.

### Selection of reports to be studied

First, one researcher (LL) picked out duplicate reports using the reference management software EndNote X6, and scanned the titles and abstracts of the citations retrieved by the selection search engine in EndNote X6 (first scanning). Then, the full texts of all potentially eligible reports were viewed together by two researchers (LL and ZL). If a report either did not meet the inclusion criteria or it met the exclusion criteria, they would move it into the appropriate folder with labels in EndNote X6. Several controversial reports were marked as either ‘‘suspicious,’’ or “waiting for the next selection.”

### Data extraction

Two researchers (LL and ZL) used the EpiData 3.1 software (The EpiData Association, Odense, Denmark) to extract and enter the findings from the final included reports by using a unified structure form. Data extracted from each study included the title, publication year, regions where RCTs were conducted, impact factor, single center/multi-center, study groups, choice of interventions, human systems, sample size and funding sources.

### Inter-evaluator reliability of ratings

Trial assessment was performed independently by 2 evaluators (LL and ZL) who had been trained in PRECIS-2. The assessments utilized a scale of 1–5 for each criterion (1 = maximal efficacy, 5 = maximal effectiveness). To test the feasibility of applying the PRECIS tool to CHM RCTs and to ensure that the criteria could be applied consistently by more than one person, we pilot-tested a draft data abstraction form using a random sample of 15 included studies prior to beginning data abstraction. The intraclass correlation coefficient (ICC) was calculated both before and after agreement. The ICC calculation formula was as follows:

$$ICC=\frac{\sum_{i=1}^{n}\left(x_{1i}-\bar{x}\right)\left(x_{2i}-\bar{x}\right)}{\left(n-1\right)s_{x}^{2}}$$

$x_{1i}$ and $x_{2i}$ represented the observed values from LL and ZL, respectively. $\bar{x}$ was the pooled mean of the two evaluators’ evaluations. $s_{x}^{2}$ was the pooled variance of all values. n was the sample size.

After this, we proposed our comments on PRECIS-2 criteria’s’ ratings operationalization and improvement. Then, we started rating all reports. Disagreements between the two researchers were discussed by the whole team and ultimately a consensus was reached.

### Statistical analysis

Ratings along the efficacy-effectiveness continuum were summarized by descriptive analysis for each time period. Previous studies have argued that a period of 3–4 years after the publication of standards is sufficient to ensure the promotion and adoption of new guidelines. [19, 20] The PRECIS tool was developed in 2009. Thus, the publication year of 2013 was used as the cut-off point. We calculated the proportion of each PRECIS criterion’s score before 2013 (including 2013) as well as after 2013. We then compared the distribution of each criterion between different date ranges using a rank-sum test. Descriptive statistical analysis and statistical inference were performed using SPSS V.18.0 (SPSS, Illinois, USA).

## CONCLUSION

To the best of our knowledge, this is the first study to investigate the impact of the introduction of PRECIS on the design and implementation of RCTs of CHM. It was found that after the implementation of PRECIS, the design and performance of RCTs of CHM did not improve, in terms of the “efficacy” criterion. We expect an improved version of PRECIS, as well as its promotion to contribute to the progress in considering “efficacy” and “effectiveness” criteria before trial designs in the future.

## SUPPLEMENTARY MATERIALS


