# Supplementary material for: The application of PRECIS-2 ratings in randomized controlled trials of Chinese herbal medicine
Source: Oncotarget. 2017 Oct 31;8(63):107002–10. doi: 10.18632/oncotarget.22204 (PMC5739791; doi:10.18632/oncotarget.22204)
Supplement: Supplementary file 1 [file oncotarget-08-107002-s001.pdf]

The application of PRECIS-2 ratings in randomized controlled trials of Chinese herbal medicine

SUPPLEMENTARY MATERIALS

MEDLINE (OVIDSP) SEARCH STRATEGY (DECEMBER 13, 2016)

1. exp Herbal Medicine/
2. exp Plants, Medicinal/
3. exp medicine, oriental traditional/
4. exp Medicine, East Asian Traditional/
5. exp Drugs, Chinese Herbal/
6. (chinese adj5 (herb* or medic* or drug*)).tw.
7. (herb* adj5 (medic* or drug*)).tw.
8. or/1-7
9. randomized controlled trial.pt.
10. controlled clinical trial.pt.
11. randomi?ed.ab.
12. placebo.ab.
13. clinical trials as topic.sh.
14. randomly.ab.
15. trial.ti.
16. or/9-15
17. exp animals/ or exp invertebrate/ or animal experiment/ or animal model/ or animal tissue/ or animal cell/ or nonhuman/
18. 16 not 17
19. 8 and 18

EMBASE (OVIDSP) SEARCH STRATEGY (DECEMBER 13 2016)

1. exp Drugs, Chinese Herbal/
2. exp medicine, oriental traditional/
3. exp Medicine, East Asian Traditional/
4. exp Herbal Medicine/
5. exp Plants, Medicinal/
6. exp herbaceous agent/
7. (chinese adj5 (herb* or medic* or drug*)).tw.
8. (herb* adj5 (medic* or drug*)).tw.
9. or/1-8
10. Randomized controlled trials/
11. Single-Blind Method/
12. Double-Blind Method/
13. Crossover Procedure/
14. (random$ or factorial$ or crossover$ or cross over$ or cross-over$ or placebo$ or assign$ or allocat$ or volunteer$).ti,ab.
15. (doubl$ adj blind$).ti,ab.
16. (singl$ adj blind$).ti,ab.
17. or/10-16
18. exp animals/ or exp invertebrate/ or animal experiment/ or animal model/ or animal tissue/ or animal cell/ or nonhuman/
19. 17 not 18
20. 9 and 19

AMED (OVIDSP) SEARCH STRATEGY (DECEMBER 13, 2016)

1. exp Herbal drugs/
2. exp Plants medicinal/
3. exp traditional medicine chinese/
4. exp Drugs, Chinese Herbal/
5. (chinese adj5 (herb* or medic* or drug*)).tw.
6. (herb* adj5 (medic* or drug*)).tw.
7. or/1-6
8. exp Randomized controlled trials/
9. exp Clinical trials/
10. exp Random allocation/
11. exp Double blind method/
12. (clin$ adj25 trial$).ti,ab.
13. ((singl$ or doubl$ or trebl$ or tripl$) adj25 (blind$ or mask$)).ti,ab.
14. exp Placebos/
15. placebo$.ti,ab.
16. random$.ti,ab.
17. exp prospective studies/
18. or/8-17
19. 7 and 18

CENTRAL SEARCH STRATEGY (DECEMBER 13, 2016)

#1. MeSH descriptor Drugs, Chinese Herbal, this term only

#2. MeSH descriptor Medicine, Chinese Traditional explode all trees

#3. MeSH descriptor Medicine, East Asian Traditional, this term only

#4. (chinese near (traditional or medicine*))

#5. herbs or herbal or herb

#6. plant or plants

#7. MeSH descriptor Phytotherapy, this term only

#8. (traditional near medicine*)

#9. (#1 OR #2 OR #3 OR #4 OR #5 OR #6 OR #7 OR #8)

#10. (random$ or factorial$ or crossover$ or cross over$ or cross-over$ or placebo$ or assign$ or allocat$ or volunteer$):ti,ab,kw

#11. #9 and #10
